# RIB OSTEOMYELITIS: A RARE COMPLICATION OF VARICELLA

**DOI:** 10.1590/1984-0462/;2019;37;4;00009

**Published:** 2019-07-04

**Authors:** Madalena Sales Luís, Filomena Cardosa, Filipa Reis, Ana Sofia Fraga, Margarida Victor, Joaquim Geraldes Santos, Paulo Calhau

**Affiliations:** a Hospital São Francisco Xavier, Hospital Center “Lisboa Ocidental”, EPE, Lisbon, Portugal.; b Hospital Garcia de Orta E.P.E, Almada, Portugal.

**Keywords:** Chickenpox, Osteomyelitis, Child, Varicela, Osteomielite, Criança

## Abstract

**Objective:**

To report a case of varicella complicated by acute osteomyelitis in order to remind of a rare and potentially serious complication of a very common pediatric disease.

**Case description:**

A previously healthy 3-month-old female infant with 10-day history of varicella was admitted to the hospital for fever, groan and prostration. The initial laboratorial evaluation was compatible with bacterial sepsis. By the third day after admission, a swelling of the seventh left rib had developed. The ultrasound and scintigraphy evaluation suggested rib osteomyelitis. Blood cultures were negative. The patient completed six weeks of antibiotics with favorable clinical, laboratorial and imaging evolution.

**Comments:**

Varicella is one of the most frequent exanthematic diseases of childhood and it is usually self-limited. The most frequent complication is bacterial infection of cutaneous lesions. Osteoarticular complications are rare, and rib osteomyelitis is described in less than 1% of cases. The main route of dissemination is hematogenic, and the most frequent etiological agent is *Staphylococcus aureus*. The prognosis is generally good and depends on early detection and antibiotic initiation.

## RESUMO

**Objetivo:**

Descrever um caso de varicela complicada de osteomielite aguda a fim de alertar para a complicação rara e potencialmente grave de uma doença muito frequente em idade pediátrica.

**Descrição do caso:**

Lactente de três meses, previamente saudável, internada por um quadro de febre, prostração e gemido, num contexto de varicela com cerca de dez dias de evolução, com avaliação inicial compatível com sepse de etiologia bacteriana. No terceiro dia de internação, observou-se uma tumefação na sétima costela esquerda. A avaliação ecográfica e cintilográfica mostraram alterações sugestivas de osteomielite de arco costal. As hemoculturas foram negativas. Recebeu antibioticoterapia por seis semanas e evoluiu favoravelmente do ponto de vista clínico, laboratorial e ecográfico.

**Comentários:**

A varicela é uma das doenças exantemáticas mais frequentes da infância, sendo habitualmente autolimitada. A complicação mais frequente é a infecção bacteriana secundária das lesões cutâneas, sendo raras as complicações osteoarticulares. O arcabouço costal é uma localização excepcional de osteomielite, descrita em menos de 1% dos casos. A principal via de disseminação é a hematogênica, e o agente mais frequente, o *Staphylococcus aureus*. O prognóstico é geralmente bom, quando a antibioticoterapia se institui precocemente.

## INTRODUCTION

Varicella is an infection caused by the varicella-zoster virus, very common in pediatric age and occurring in the first decade of life in 90% of the cases.^1^ It is usually benign and most patients recover without sequelae. However, there are more vulnerable groups in the pediatric age, such as young infants and immunocompromised children.^2^

Infection of the skin and soft tissues is frequent and occurs in 2‒5% of cases. Bacteremia/sepsis, pneumonia, cerebellar ataxia, encephalitis, hepatitis, pancreatitis, myocarditis, nephritis and thrombocytopenia are less frequent complications, while musculoskeletal complications (septic arthritis, acute osteomyelitis, pyomyositis and necrotizing fasciitis) are rare.^3^ Osteomyelitis accounts for 0.02% of complications secondary to varicella.^4^

The objective of this report is to describe a case of varicella complicated by acute osteomyelitis in order to raise awareness about a rare complication of a very frequent disease in pediatric age.

## CASE REPORT

Three-month-old female infant, admitted for social reasons. Fourth daughter of non-consanguineous parents; mother with cognitive deficit. Born of 36-week pregnancy complicated by gestational diabetes in the third trimester. Caesarean delivery due to dystocia. Apgar Index=8/9; birth weight of 2,650 g (Percentile_3-15_), length of 45.5 cm (Percentile_3_), head circumference of 33.2 cm (Percentile_15_). Admitted in the first five weeks of life to the neonatal intermediate care unit due to respiratory failure, asymptomatic hypoglycemia, feeding difficulty and by social reason. Second admission to hospital at two months old, lasting 48 hours, for acute bronchiolitis caused by respiratory syncytial virus (RSV). Adequate weight-bearing growth and psychomotor development.

The infant was taken to the pediatric emergency room for a clinical picture of 48 hours of fever (maximum axillary temperature 38.5°C), prostration and groaning, in the context of varicella, with the first skin lesions identified for about ten days. Upon examination at the emergency room, the infant had fever, was groaning, normal fontanel, blood pressure of 94/54 mmHg, heart rate at 150 bpm, good perfusion, eupneic. Numerous skin lesions in crust throughout the body, with signs of secondary bacterial infection on the scalp. Remaining examinations showed no alterations. Laboratorial results were (Table 1): hemoglobin 76 g/L, leukocytes 45,7×10^9^/L (neutrophils 51%), platelets 588×10^9^/L, C-reactive protein (CRP) 18.8 mg/dL, glycemia, serum transaminases and blood gas analysis were normal, two negative blood cultures, urinalysis without abnormal findings, sterile urine culture, normal chest radiography.

**Table 1:** Progression of laboratory evaluation.

Lab standards [reference value]	Admission	D1	D4	D10	Six weeks of therapy
Hb (g/dL) [100-145]	76	75	79	86	92
MGV (fL) [70-95]	76.6	77.1	76	76.9	76
MGH (pg) [24-30]	23.7	24.1	24.2	26.2	24
Platelets (*10^9^/L) [130-400]	588	664	661	625	234
Leukocytes (*10^9^/L) [4-11]	45.7	42.3	13.4	14.8	16.1
Neutrophils (*10^9^/L/%) [1,9-8/40-74]	23.3/51	25.1/ 29	2.01/15	2.8/ 18.8	1.7 / 11
C-reactive protein (mg/dL) [<0,2]	18.8	26.4	4.8	0.3	0.3
ESR (mm/1st hour) [<16]	--	--	80	35	10

Hb: hemoglobin; MGV: mean globular volume; MGH: mean globular hemoglobin; ESR: Erythrocyte sedimentation rate.

The patient was admitted with diagnostic hypothesis of sepsis, probably secondary to impetiginized varicella. Intravenous antibiotic therapy with ceftriaxone (100 mg/kg/day) and topical (fusidic acid) was initiated.

On the third day of hospitalization, a swelling was identified in the left 7th rib, at the nipple line, with hard consistency and poorly defined limits, apparently painful to palpation and no other local signs of inflammation.

**Figure 1:**
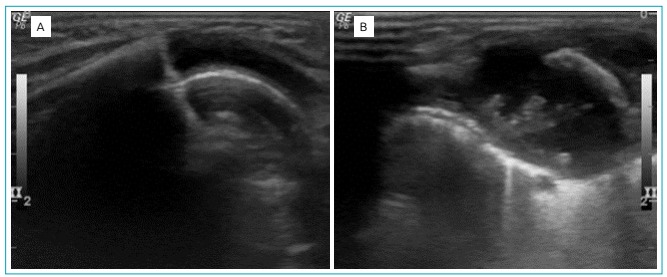
Ultrasound of soft parts of right and left costal frameworks, respectively. (A) To the right, unchanged; (B) to the left, a volumetric increase of the costochondral joint is seen, with free intra-articular fluid and irregularity of the anterior end of the costal arch.

**Figure 2:**
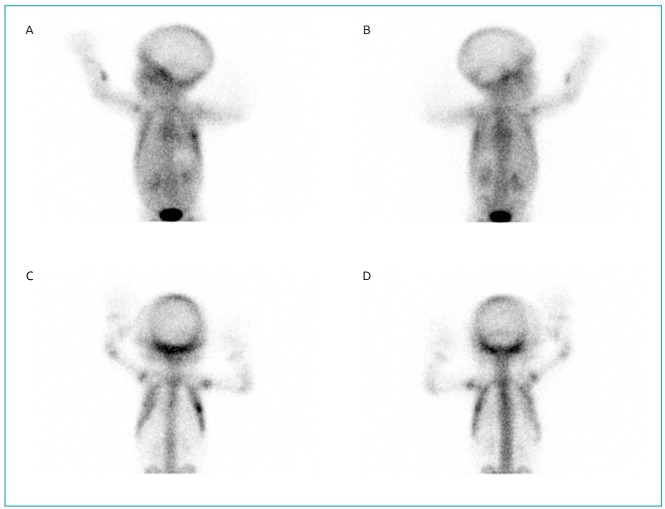
Bone scintigraphy - 99mTC-HMDP radiopharmaceutical. (A) and (B) initial images: focal hyperaemia in middle anterior region of left costal scaffold; (C) and (D) late images: hypercaptation of radiopharmaceutical in topography corresponding to the focus of hyperemia identified in the diffusion phase and area of hypofixation of the radiopharmaceutical upon topography of respective costochondral joint.

On the following day, laboratorial tests were repeated (Table 1): hemoglobin 79 g/L, leukocytes 13.4×10^9^/L (neutrophils 15%), CRP 4.8 mg/dL, sedimentation rate of 80 mm/1st hour. Chest radiography did not show changes in costal arches. Ultrasound of soft tissues (Figure 1) showed volumetric increase of the costochondral joint with free intra-articular fluid and irregularity of the costal arch’s anterior end. Three-phase 99mTc-HMDP bone scintigraphy (Figure 2) showed focal hyperemia in the left anterior mid-arch of the left costal arch and, in late images, high uptake of the radiopharmaceutical in topography corresponding to the focus of hyperemia identified in blood diffusion phase and area of hypo fixation of the radiopharmaceutical upon respective costochondral joint topography, aspects compatible with inflammatory/infectious pathology. The distribution of the radiopharmaceutical was normal in the rest of the skeleton.

**Figure 3:**
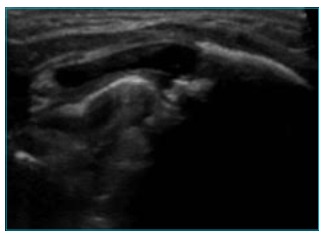
Ultrasound of left costal arch soft tissues of the. An irregularity of the anterior extremity of the arch is observed, although it shows sclerotic borders of apparently continuous form, with aspect suggesting consolidation.

During hospitalization, the infant was always hemodynamically stable, with apirexia from the third day and progressive improvement of general condition and cutaneous lesions. Ten-day therapy with ceftriaxone was completed, at which time the infant was submitted to laboratory reassessment (Table 1): hemoglobin 86 g/L, leucocytes 14.8×10^9^/L (neutrophils 19%), CRP 0.3 mg/dL and sedimentation rate at 35 mm/1st hour. Oral amoxicillin-clavulanic acid (70 mg/kg/day) was initiated, completing six weeks of antibiotic therapy, presenting at the time of resolution of the palpable swelling sedimentation rate at 10 mm/1^st^ hour, CRP lower than 0.1 mg/dL (Table 1) and control ecography showing irregularity of the costal arch’s anterior extremity, although sclerotic borders of apparently continuous form and aspects suggestive of consolidation were seen (Figure 3).

Follow-up was maintained with appointments in Pediatrics, and immunity was then tested with determination of immunoglobulins, resulting normal. There were no intercurrences in the following year after hospital admission.

## DISCUSSION

Acute osteomyelitis is relatively uncommon in pediatric age, but is one of the leading invasive bacterial infections resulting in prolonged hospital stay and antibiotic therapy. In developed countries, osteomyelitis has an estimated incidence of 8:100 thousand children/year, while in less developed countries values are considerably higher. It occurs mostly in males, at a proportion evaluated at 2:1.^5^ ^,^ ^6^ ^,^ ^7^

*Staphylococcus aureus* is the most frequent etiological agent, followed by *Streptococcus pyogenes* and *Streptococcus pneumoniae*. Salmonella infection is common in developing countries and in patients with sickle cell disease. *Kingella kingae* has become increasingly important, particularly among children under four years of age. Tuberculous and fungal etiologies have become more expressive in less developed countries, often in immunosuppressed patients with disseminated and multisystemic disease. In the cases of varicella-associated osteomyelitis, several cases reported by literature indicate *S. pyogenes* as the most frequently implicated bacterium, followed by *S. aureus*.^8^ ^,^ ^9^

In children, the hematogenic route is the most common, but the infectious agent, almost always bacterial, can also reach the bones by direct inoculation after the evolution of a traumatic lesion, or by local dissemination through a contiguous infection (cellulitis, empyema, pneumonia or septic arthritis). In cases associated with varicella, secondary bacterial infection occurs after rupture of the protective barrier caused by vesicular skin lesion and itching lesions, which may lead to bacteremia and, indeed, by changes in immune functions induced by the virus. In *S. pyogenes* secondary infection, the enzymes hyaluronidase and streptolysin, produced by this bacterial strain, appear to facilitate the penetration of bacteria into deeper tissues.^9^ ^,^ ^10^

Osteomyelitis is unifocal in most cases, but may be multifocal at any age, especially in newborns.^10^ It occurs mainly in metaphysis of long bones, particularly the femur, tibia and humerus. The costal framework is a rare localization, being described in less than 1% of cases. Following the hematogenous spread, the preferred bone locations for the infection to occur are areas of increased vascular supply, where bone is metabolically more active. In the costal framework, it most often corresponds to the costochondral junction (anterior) and to the costovertebral angle (posterior).^11^

A description of the first case of costal osteomyelitis by Chauvenet et al. dates 1885. More recently, a review of the literature described 57 cases of osteomyelitis in the costal framework in pediatric age, corresponding to a period of 48 years (1963‒2011). The mean age was 6.6 years, with no predominance of sex; fever was reported in 36% of cases; multifocal involvement occurred in 9%; the predominant etiological agent was *S. aureus* (36%); the hematogenous pathways and contiguous dissemination were acknowledged in 52 and 42% of the cases, respectively; drainage was performed in 94% of patients and bone resection in 75% of them.^11^

The clinical presentation of osteomyelitis is almost always acute, and may include fever, functional impotence, pain and other inflammatory signs of the involved area. However, its beginning, progression and course may be more insidious, which may condition a significant delay in diagnosis. Osteomyelitis is classified as acute when, at the time of diagnosis, it is reported to last less than two weeks, as subacute in cases lasting between two weeks and three months, and as chronic in longer periods.^8^

The differential diagnosis of lytic lesions acquired in costal arches should include trauma, metabolic alterations (such as rickets and hyperparathyroidism), iatrogeny (in the context of prostaglandins use) and neoplastic disease; hypotheses are excluded by anamnesis, objective examination, clinical, analytical and echographic evolution.^12^

CRP, procalcitonin and erythrocyte sedimentation rate are highly sensitive and useful inflammatory parameters in follow-up, namely when it comes to the evaluation of response to therapy and detection of complications.^13^ ^,^ ^14^ ^,^ ^15^ In this case, CRP and erythrocyte sedimentation rate were initially high, but their evolution became rapidly favorable.

Image alterations identified in children with varicella-associated osteomyelitis overlap with those observed in other cases of bacterial osteomyelitis.^16^ Plainly pathological radiological aspects of cortical reaction and bone destruction are not usually identified in the first two weeks of symptoms, which is why a chest radiography at the time of hospital admission is the standard (as noted in the case reported here), but it should not therefore rule out the diagnosis of osteomyelitis, while it has the additional value of excluding a bone fracture or tumor.^8^

Bone scintigraphy, necessarily multiphasic, presents changes very early (24‒48 hours), has high sensitivity and the enormous advantage of not requiring sedation, an aspect that was taken into account in this child.^16^ In the case described, in addition to the usual early increase of the radiopharmaceutical activity in the affected bone site, increased in the late phase of the examination, an area of hyper uptake was reported, which, according to some authors, may be a warning sign of a more aggressive disease, with reduced perfusion and even bone necrosis.^17^

Magnetic resonance imaging is, however, the most sensitive and specific test for the diagnosis of osteomyelitis, allowing early identification (24‒48 hours) of changes such as spinal edema, which occurs in the early stages of metaphyseal infection. The extent of involvement and disease activity in subacute or chronic cases are also important contributions of this imaging exam.^18^ ^,^ ^19^

Ultrasonography can be very informative, especially in infants, when it reveals very early (48 hours of disease) periosteal thickening, presence of subperiosteal fluid/abscess, alterations of adjacent soft tissues, which allows the monitoring of its evolution, as shown in this case, with the advantage of being non-invasive.^20^

Etiological diagnosis is only possible in cases of positive blood culture (which occurs in only about 40% of cases) or in microbiological examination of metaphysis aspirate or subperiosteal pus in cases of costal osteomyelitis.^8^ ^,^ ^11^ In our patient, blood cultures were negative, and good clinical, imaging and clinical evolution made any surgical aspiration/drainage unnecessary, so the etiology was not determined.

The initial treatment of acute osteomyelitis is empirical, with antibiotic therapy necessarily covering *S. aureus* and other gram-positive agents. In this case, the evidence, on the third day of hospitalization, of a clinical picture suggestive of varicella-related costal osteomyelitis, with negative blood culture and good thermal curve response, resulted in maintenance of initial therapy of intravenous ceftriaxone. The subsequent clinical and laboratorial evolution allowed to use oral route, with institution of amoxicillin and clavulanic acid on the 11th day of treatment. Some authors advocate an even shorter duration of intravenous antibiotic therapy for uncomplicated acute osteomyelitis.^21^ ^,^ ^22^ The therapy totaled six weeks, according to recommendations of four to six weeks duration in the literature.^23^ Some authors recommend shorter treatment schedules, which enhances efficacy, lowers costs and reduces the risk of bacterial resistance.^8^

In cases of early diagnosis, like our patient’s, conservative medical treatment of acute osteomyelitis for children is effective in about 90% of the cases.^24^ Curiously, in the literature review, the percentage of interventions in cases of rib osteomyelitis was extremely elevated.^11^ In acute cases with associated abscess, and cases of intraosseous abscess of subacute or chronic osteomyelitis (Brodie’s abscesses), surgical drainage is imperative.^8^

Currently, osteomyelitis is rarely fatal in developed countries, and functional prognosis is usually favorable, particularly when there’s early access to health care, diagnosis, hospitalization and antibiotic therapy.^7^ ^,^ ^22^

The case described is a rare complication of varicella in an exceptional location. The rapid diagnosis and immediate institution of therapy were determinant for the good clinical evolution.
